# Among-individual variation in thermal plasticity of fish metabolic rates causes profound variation in temperature-specific trait repeatability, but does not co-vary with behavioural plasticity

**DOI:** 10.1098/rstb.2022.0488

**Published:** 2024-02-26

**Authors:** Tommy Norin, Lauren E. Rowsey, Thomas M. Houslay, Connor Reeve, Ben Speers-Roesch

**Affiliations:** ^1^ DTU Aqua: National Institute of Aquatic Resources, Technical University of Denmark, Henrik Dams Allé 202, 2800 Kgs. Lyngby, Denmark; ^2^ Department of Biological Sciences, University of New Brunswick, Saint John, New Brunswick, Canada E2L 4L5; ^3^ Centre of Ecology and Conservation, College of Life and Environmental Sciences, University of Exeter, Penryn Campus, Penryn, Cornwall, TR10 9FE, UK; ^4^ Department of Biology, Carleton University, Ottawa, Ontario, Canada K1S 5B6

**Keywords:** phenotypic plasticity, reaction norm, performance curve, physiology, growth rate, pace-of-life syndrome

## Abstract

Conspecifics of the same age and size differ consistently in the pace with which they expend energy. This among-individual variation in metabolic rate is thought to influence behavioural variation, since differences in energy requirements should motivate behaviours that facilitate energy acquisition, such as being bold or active in foraging. While there is evidence for links between metabolic rate and behaviour in constant environments, we know little about whether metabolic rate and behaviour change together when the environment changes—that is, if metabolic and behavioural plasticity co-vary. We investigated this using a fish that becomes dormant in winter and strongly reduces its activity when the environment cools, the cunner (*Tautogolabrus adspersus*). We found strong and predictable among-individual variation in thermal plasticity of metabolic rates, from resting to maximum levels, but no evidence for among-individual variation in thermal plasticity of movement activity, meaning that these key physiological and behavioural traits change independently when the environment changes. The strong among-individual variation in metabolic rate plasticity resulted in much higher repeatability (among-individual consistency) of metabolic rates at warm than cold temperatures, indicating that the potential for metabolic rate to evolve under selection is temperature-dependent, as repeatability can set the upper limit to heritability.

This article is part of the theme issue ‘The evolutionary significance of variation in metabolic rates’.

## 1. Introduction

Individual animals of the same species and size can vary profoundly in the rate with which they consume oxygen and expend energy (i.e. their metabolic rate), with a two- to threefold range in metabolic rates among same-sized individuals [1,2]. This among-individual variation in metabolic rate is generally consistent (i.e. repeatable; [3–5]) and related to key organismal traits such as behaviour and growth [6–9]. For example, individuals with relatively high metabolic rates have been found to out-grow their low-metabolic-rate conspecifics when food is plentiful and predictable [10,11], likely due to more efficient food processing associated with higher metabolic rates and larger meals [12–14], whereas low-metabolic-rate individuals can out-grow their high metabolic rate counterparts under restricted or unpredictable food availability [3,15], including in the wild [16,17].

Variation in metabolic rate among individuals has also been linked with behavioural variation, so that, within a given environment, individuals with the higher resting (maintenance) metabolic rates—termed basal metabolic rate (BMR) for endotherms or standard metabolic (SMR) for ectotherms—are usually more active, aggressive, dominant and/or prone to risk-taking [6–9]. Most of this behavioural variation has been linked to variation in BMR or SMR, but variation among individuals in other metabolic traits, such as the aerobic maximum (active) metabolic rate (MMR) and aerobic scope (AS, the difference between MMR and BMR or SMR) also is correlated with behavioural variation. For example, individuals with relatively high AS have been shown to take up leading positions in swimming fish schools [18,19] and high-AS individuals have also been reported to be more aggressive and dominant [20].

These links between metabolic rate, growth and behaviour have been proposed to constitute a broader slow–fast life-history continuum [21], or pace-of-life syndrome [22]. While there is compelling empirical support for slow–fast pace-of-life histories that involve metabolic rate [23–25], the existence of a universal pace-of-life syndrome is equivocal [26], possibly because metabolic rate and behaviour may be correlated in some, but not all, environmental contexts, as found for suites of behavioural traits [27]. Since organisms can change their phenotype in response to a change in the environment through phenotypic plasticity, the lack of relationships between metabolic rate and behaviour in some contexts could be due to differences in the degree of plasticity of metabolic and behavioural traits, but the extent to which this is the case is largely unknown (see [5,28–30]). In other words, we do not know the extent to which phenotypic plasticity in metabolic rate and behaviour co-vary within individuals when the external environment changes. As phenotypic plasticity is usually believed to be adaptive ([31,32], but see [33–35]) and to increase resilience to climate change [36], variation among individuals in phenotypic plasticity could facilitate adaptation to rapid environmental changes. On the other hand, it is possible that key metabolic and/or behavioural traits trade off in their plasticity, such that individuals can be plastic in one trait at the expense of plasticity in another, as there may be costs and restrictions to plasticity that prevent individuals from being infinitely plastic [37,38]. Given that metabolic rate is generally found to be heritable [39], can be under selection [24,25,40,41] and can evolve rapidly [23], understanding how and in what contexts metabolic rate (co)varies with other key organismal traits such as growth and behaviour is important for understanding how these traits may (co)evolve under environmental change.

Here, we used a marine fish, the cunner (*Tautogolabrus adspersus*), which becomes dormant in winter and is known to strongly reduce its activity when the environment cools [42,43], to investigate whether there is: (1) evidence for among-individual variation in phenotypic plasticity of resting and maximum metabolic rates, growth and behavioural (voluntary swimming) activity across a seasonally realistic range and change in temperature; and (2) evidence for co-variation in plasticity between these key organismal traits. We quantified variation in phenotypic plasticity as variation in trait thermal sensitivity, with thermal sensitivity quantified as the slope of a regression of the phenotypic trait of interest across temperatures, a so-called thermal reaction norm [35,37].

## 2. Methods

### (a) Animals and holding conditions

Juvenile cunner (*Tautogolabrus adspersus*; *n* = 75) were obtained from Cooke Aquaculture captive breeding programme at the Huntsman Marine Science Centre (St. Andrews, New Brunswick, Canada) in January 2018 and transported to the University of New Brunswick (UNB), Saint John, Canada. These fish were F1 offspring born and reared in 2017 from wild-caught parents from Saint Mary's Bay, Nova Scotia. At UNB, the fish were held in an aquarium room in five 60 L glass aquaria (15 fish per aquarium) receiving 33 ppt salinity seawater that recirculated through active biomedia and a UV filter, was mechanically filtered, aerated and occasionally replaced. Each aquarium had three pieces of opaque PVC pipe and two pieces of curved plastic mesh for sheltering and enrichment. Water temperature was initially maintained at 14 ± 0.5°C using a 1/3 HP Arctica chiller (JBJ Chiller, St. Charles, MO, USA). The fish were fed commercial pellets (Gemma Diamond 1.8 mm; Skretting, St. Andrews, NB, Canada) once daily on weekdays.

In preparation for experiments, on 6 May 2018, the 75 cunner were tagged for individual recognition using unique colour combinations of Visible Implant Elastomer (Northwest Marine Technology; Anacortes, WA, USA) injected subcutaneously with a fine 30 G needle. At this point, the fish weighed 7.27 ± 1.75 g (mean ± s.d. body mass; range 3.98 to 10.48 g) and were 82 ± 6.5 mm long (mean ± s.d. total length; range 70–92 mm).

### (b) Study overview

The study was aimed at quantifying (co)variation in the metabolic and behavioural thermal sensitivity (phenotypic plasticity) of individual cunner as they were gradually cooled from their initial acclimation temperature of 14°C to 2°C at an average rate of 0.12°C/day. This resembles the seasonal temperature change from early autumn to winter in the species' natural distribution range in the Canadian Maritimes (e.g. 0.10–0.12°C/day from September to December for locations Shediac and Bathurst, New Brunswick, and Parrsboro and Hantsport, NovaScotia, calculated from www.seatemperature.org).

To quantify metabolic and behavioural plasticities, we measured metabolic rates and behaviours of the same 75 individuals at 14, 11, 8, 5 and 2°C during cooling in the lab from mid-May to late August 2018. We also recorded the fishes' body masses and thus their growth rates across the five temperatures. Cooling was done gradually in the fishes' holding aquaria by reducing the chiller setpoint by 0.5–0.6°C (1°F) every 4 days, except during the five 7-day measurement periods when temperature was kept constant (within 0.5°C) at the target temperatures while the metabolic rates and behaviours of the 75 fish were being assessed.

Behaviour was quantified as voluntary movement activity (average swimming speed) by video recording fish in individual behavioural arenas followed by automated tracking of their movements, as detailed below. We recorded the fish during both day and night (under near-infrared light, 930 nm) and quantified both day- and night-time activity.

Metabolic rates were estimated as oxygen uptake rates measured using best-practices intermittent-closed respirometry [44,45]. Fish were fasted for approximately 23 h before initiation of oxygen uptake rate measurements. Both the aerobic maximum (active) metabolic rate (MMR) and the standard (resting) metabolic rate (SMR) were quantified, as detailed below, which allowed for additional calculations of aerobic scope (AS), the absolute difference between MMR and SMR.

Complementary to this first and relatively slow cooling study (hereafter ‘slow cooling experiment’), a second follow-up study using the same fish was performed, in which we quantified behavioural thermal sensitivity during more rapid cooling (hereafter ‘fast cooling experiment’) for comparison with behavioural plasticity during the slow cooling experiment. We did this by re-acclimating the fish to 14°C for two months, following completion of the slow cooling experiment, before cooling them again to 2°C at an average rate of 2°C per day. Cooling was done directly in the behavioural setup, with the fish kept and fed there for the 7 days it took to get from 14 to 2°C. Behaviour was quantified as for the slow cooling experiment, but for every 2°C (i.e. daily). As we fed the fish in their behavioural arenas, we also quantified daily food intake in addition to movement activity, as detailed below.

A timeline for the study can be found in the electronic supplementary material, figure S1.

### (c) Behavioural setup

The behavioural setup consisted of 16 behavioural arenas, which were transparent plastic boxes measuring 20.2 × 15.6 × 9.7 cm (l × w × h; water depth 8 cm), each receiving aerated and temperature-controlled seawater from a sump with active biomedia. Water exited each arena through an overflow hole and drained into a transparent (acrylic) outer tank from which it returned to the sump before being recirculated back to the arenas. Water in the setup was changed between each behavioural trial. Each arena had an 8 cm-long shelter (opaque PVC pipe) along the long side of the arena, with one end pushed up against the end-wall (i.e. fish could only enter and exit the shelter from one end). The outer tank containing the behavioural arenas was placed on a false floor of white translucent PVC sheet, which was illuminated from below with near-infrared lights, creating a sharp silhouette of the fish and allowing for both day- and night-time video recordings using a set of two near-infrared-sensitive USB cameras (IDS Imaging Development Systems GmbH, Obersulm, Germany) mounted above the behavioural arenas and connected to a laptop, with each camera recording eight arenas. For the first slow cooling experiment, the fish were video recorded for 1 h during both day (10.00–11.00) and night (01.00–02.00), while for the follow-up fast cooling experiment, fish were recorded for 6.5 h during the day only (9.00 to 15.30). A detailed description of the behavioural setup used here, with illustration and photos, can be found in Reeve *et al*. [43] and associated supplementary information (their electronic supplementary material, figure S1).

### (d) Respirometry setup

The respirometry setup consisted of 16 cylindrical respirometry chambers submerged in an ambient water bath containing approximately 60 L of aerated seawater maintained at the experimental temperature using a thermostatted water bath. The respirometry chambers were 165 mm long glass tubes of 32 mm inner diameter, with an acrylic endcap at each end that sealed against the glass tube with a rubber O-ring. The free internal space inside each respirometry chamber between endcaps measured 128 mm. Each endcap had two stainless steel tube connectors from which each respirometry chamber was connected to two sets of PVC tubing (Masterflex Tygon E-Lab); one set recirculated water continuously in a closed loop through the chamber and past an optical oxygen probe (mounted in an acrylic probe holder) by means of a peristaltic pump (Masterflex L/S with Masterflex Tygon E-LFL 2-stop tubing), while the other set was used to intermittently flush the respirometry chamber with aerated water from the ambient tank by means of an aquarium pump (Eheim Compact 300) controlled by a relay timer. Two 8-channel peristaltic pumps were used to recirculate water through the 16 respirometry chambers, and four flush pumps spilt four-ways (using a regulable aquarium airline manifold) were used to flush the 16 chambers.

The automated intermittent flushing of the respirometry chambers allowed for repeated recordings of fish oxygen uptake rates during the closed periods when the flush pumps were off and the respirometry chambers effectively sealed, while replenishing water oxygen levels and removing metabolic waste products during flush periods. Schematics of a respirometry setup like the one used here can be found in Killen *et al*. [45] (their fig. 1).

**Figure 1.**
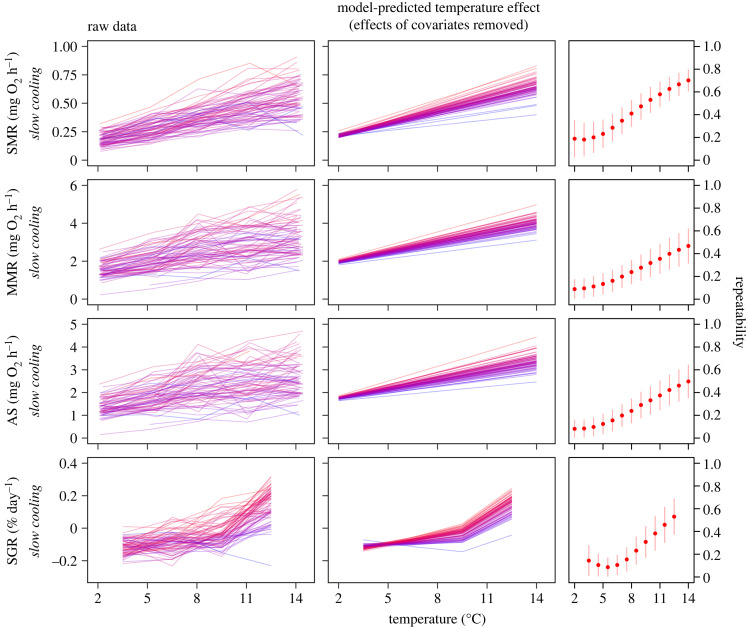
Metabolic and growth rates of individual cunner (coloured lines) cooled slowly from 14 to 2°C at a rate of 0.12°C day, which represents the seasonal temperature change in the ocean during autumn. Panels show raw data, model-predicted temperature effects (thermal reaction norms) from the random slopes models after controlling for covariates (primarily body mass, but also minor effects of respirometry chamber and/or holding aquarium) and conditional (context-specific) repeatability estimates for standard metabolic rate (SMR), maximum metabolic rate (MMR), aerobic scope (AS) and specific growth rate (SGR). Note that SGRs were measured between each set of two consecutive temperatures and are therefore aligned with the mean temperature of each growth interval (e.g. SGR at 12.5°C is the growth that occurred during the time period when fish were cooled from 14 to 11°C). Lines representing individual fish are coloured-coded within traits in translucent blue to red from the lowest to highest model-predicted trait values at 8°C, the model intercept temperature; darker regions are due to overlap. For repeatability, red dots and red vertical lines are mean conditional repeatability estimates and their 95% confidence intervals (CIs), respectively, for each trait.

The total volume of a respirometry chamber (including the recirculation loop and oxygen probe holder) was 126.5 mL on average, ranging from 124 to 129 mL depending on recirculation loop tube length. The respirometry chamber itself was 116 mL. The 16 optical oxygen probes were connected to four 4-channel optical oxygen meters (FireStingO2; PyroScience GmbH, Aachen, Germany) that recorded water oxygen concentration (mg/L) every 2 s onto a laptop running PyroScience Pyro Oxygen Logger software.

### (e) Experimental protocol: slow cooling experiment

On a given experimental day, a batch of 15 fish were removed by hand netting from their holding aquaria in the afternoon (between approx. 15.00 and 16.00) and moved in a water-filled bucket to the behavioural setup in a nearby room, where the fish were distributed into 15 of the 16 behavioural arenas kept at the target experimental temperature (the same as the temperature in the fishes' holding aquaria; i.e. 14, 11, 8, 5 or 2°C ± 0.5°C). The fish were then left undisturbed until the following day, with the camera software automatically initiating video recordings of the fish in their behavioural arenas from 01.00–02.00 and 10.00–11.00.

On the following day, the fish were removed from their behavioural arenas by hand at around 12.00 and moved to the nearby respirometry room in a water-filled bucket, where they were released into a temporary holding tank kept at the experimental target temperature. To elicit MMR, the fish were then individually and vigorously chased to exhaustion by hand in a round tub with approximately 20 L of water at the target temperature, after which the fish were immediately transferred to a respirometry chamber. Fish were deemed exhausted when they became unresponsive to having their tail pinched and being turned upside-down, which generally occurred within approximately 1 min at all temperatures, with the exact time to exhaustion noted for each individual fish. As soon as a fish was placed in the respirometry chamber and the chamber closed—usually within 15 s from cessation of chasing—the flush pump was manually turned off, initiating the first closed period of the respirometry trial, during which the fish's oxygen uptake rate was recorded over the following 3–11 min, with longer recordings at colder temperatures.

The next individual fish in the batch of 15 fish was then chased to exhaustion during the subsequent flushing of the respirometry chambers, followed by transferring that fish to its chamber and recording its oxygen uptake. This procedure was repeated until all 15 fish were in the respirometry chambers, after which the automated intermittent cycling of the closed and flush periods was initiated on the relay timer. The flush periods were 4 min at all temperatures, while the closed periods were longer at colder temperatures due to slower fish respiration rates, ranging from 5 min at 14°C to 21 min at 2°C. The fish were then left undisturbed for approximately 17–18 h (until approx. 09.00 the following day), producing between *ca* 117 and 42 individual recordings of oxygen uptake rates between 14 and 2°C, respectively, from which each fish's SMR was determined, as detailed below. The fish were then removed from their respirometry chambers, weighed and returned to their holding aquaria, and three recordings of background (microbial) oxygen uptake rates were taken in all respirometry chambers.

After background respiration recordings, the entire respirometry setup was cleaned by running diluted household bleach (approx. 1 : 100 bleach : water) through the system for approximately 20 min, except for the oxygen probes that were removed before bleaching and wiped off with 70% ethanol. When this procedure was finished, everything was rinsed by draining and refilling the setup with freshwater three times. The setup was then refilled with seawater and brought to the target experimental temperature in preparation for the next batch of 15 fish. This thorough cleaning procedure ensured that background respiration was always absent at the start of each respirometry trial.

The next batch of 15 fish then went through the same procedure until all 75 fish had been measured for behaviour and metabolic rates at the respective target temperature, which took 7 days. Then, the temperature in the fish-holding aquaria was gradually reduced towards the next target temperature (as described above) and the entire procedure repeated. This protocol was repeated until the metabolic rates and behaviours of the 75 fish had been repeatedly measured at the five target temperatures of 14, 11, 8, 5 and 2°C.

### (f) Experimental protocol: fast cooling experiment

On a given experimental day, a batch of 15 fish (the same individuals as for the slow cooling experiment) were removed by hand netting from their 14°C holding aquarium in the early morning and moved in a water-filled bucket to the behavioural setup kept at 14°C. Video recordings of the fish were then initiated at approximately 9.00 for 6.5 h at 14°C, after which temperature in the behavioural setup was decreased by 2°C by reducing the chiller setpoint in the afternoon and allowing the behavioural setup to cool over the following hour to the next target temperature of 12°C. On the following day at 09.00, the fish were video recorded for another 6.5 h at this new target temperature of 12°C, and this protocol of cooling and video recording was continued for every 2°C until the fish had reached and been recorded at 2°C, which took 7 days. The fish were then removed from the behavioural setup and the next batch of 15 fish introduced, and the protocol was repeated until all fish had been recorded.

In addition to video recording the fish for behavioural assessments, food consumption of each fish was assessed daily at each temperature by feeding the fish 10 pellets each (corresponding to approx. 0.5% of the average fish body mass) at approximately 08.30 in their behavioural arenas and noting how many pellets each fish had eaten by the end of the day.

### (g) Raw data analyses

Fish behaviours were quantified from the recorded videos using automated ToxTrac tracking software (v. 2.84), as detailed in Rodriquez *et al*. [46]. We quantified the total distance moved by each fish in their individual behavioural arenas at day and night (day only for the fast cooling experiment) and divided this by the total duration of the behavioural trial to get average day- and night-time swimming speeds (mm/s).

Oxygen uptake rates (mg/h), as proxies for metabolic rates, were calculated from the respirometry recordings by multiplying the slope for the decrease in oxygen concentration over time (mg/L/h) during each closed period of the respirometry trial by the volume of the respirometry chamber minus fish volume (L). Fish volume was determined by weighing the fish and assuming a fish density of 1 kg/L.

Before calculations of MMR and SMR, all fish oxygen uptake rates were adjusted for background (microbial) oxygen uptake. The background oxygen uptake was estimated by taking the background respiration rates recorded in the one empty (blank) chamber running in parallel with the 15 chambers with fish and combining it with the mean of the three background respiration recordings taken in each respirometry chamber after the fish had been taken out. This estimation was done by assuming that the temporal development (shape) of background respiration in the blank chamber running in parallel with the fish was representative of the shape in all other chambers with fish, and this background respiration curve was then progressively adjusted up to intersect with the mean of the three post-fish background recordings for each individual chamber. The progressive adjustment started from the point when background respiration became significantly different from zero in the blank chamber running in parallel with the fish. The resulting background oxygen uptake rates for each closed period in each individual respirometry chamber were then subtracted from the corresponding raw fish oxygen uptake rates to yield the final oxygen uptake rates.

Following adjustments for background respiration, the first measurement of oxygen uptake rate after the exhaustive chase was taken as an estimate of the fish's MMR. Standard metabolic rate was calculated from the oxygen uptake rates over the subsequent approximately 17–18 h of over-night respirometry by first identifying the lowest 10% of these oxygen uptake rates, followed by excluding any outliers and then calculating the mean of the remaining oxygen uptake rates within the initial lowest 10%. An oxygen uptake rate measurement was considered an outlier if the *R*^2^-value for the slope of the decrease in oxygen concentration over time used to calculate it fell below two standard deviations from the overall mean *R*^2^ for all slopes for that fish in the respirometry trial.

Fish growth rates were calculated in % per day (specific growth rate, SGR), as (ln(BM_final_) − ln(BM_initial_)) / days × 100, with BM_initial_ and BM_final_ being the body masses of the fish at an initial and final measurement, respectively, and days being the time in days between these measurements. Growth rates were calculated between each set of two consecutive temperatures (i.e. between 14 and 11°C, 11 and 8°C, 8 and 5°C, and 5 and 2°C), resulting in four measurements of SGR across the slow cooling experiment. For statistical analyses and presentation of data, these SGRs were aligned with temperatures in the middle of their growth interval (i.e. 12.5, 9.5, 6.5 and 3.5°C).

### (h) Statistical analyses

Data were analysed in R v. 4.3.0 [47] using generalized linear mixed models in the package MCMCglmm [48]. For the slow cooling study, data for two of the 75 individual cunner were excluded from the statistical analyses; one of these two individuals was completely excluded from the data set because it was euthanized halfway through the experiment, as it had become thin and sickly looking. For the other individual, only the metabolic rate data at 8°C were excluded, as a leak in this fish's respirometry chamber (a hole in the peristaltic pump recirculation tubing) had developed during this respirometry run, making the metabolic rate estimates unreliable. Of the original 75 fish, 72 were used in the follow-up fast cooling experiment; in addition to the one fish that had been euthanized, two fish had died for unknown reasons in-between the two experiments. Of these 72 fish, three were excluded from analyses because we were unable to video track them for behaviour due to poor fish-to-background contrast, resulting in a final sample size of 69 fish for the fast cooling experiment. The full datasets (with all individuals, including those excluded for statistical analyses) and annotated analysis R script are available in the electronic supplementary material.

Each trait of interest—SMR, MMR, AS, SGR, day-time activity, night-time activity (slow cooling experiment only) and food intake (fast cooling experiment only)—was first analysed in separate univariate models with the trait of interest as a response variable and centred temperature, scaled body mass, and their interaction as fixed-effects predictor variables; the random effects included were individual fish identity, holding aquarium, and either respirometry chamber for metabolic rate traits or behavioural arena for behavioural traits. Fixed effects were considered significant if the 95% highest posterior density credible intervals (95% CIs) excluded zero. We also report Markov Chain Monte Carlo *p*-values (pMCMC; the probability that the posterior distribution includes zero). The interaction between temperature and body mass was dropped from the models if it was not significant. To test for evidence of variation in phenotypic plasticity among individuals in response to temperature (i.e. among-individual variation in thermal sensitivity, or so-called reaction norm slopes), we ran a random intercepts model as well as a random slopes model for each trait of interest and compared these using the deviance information criteria (DIC). For DICs in general, a difference in DICs (ΔDIC) between 5 and 10 represents substantial support for a model with the lowest DIC, while a ΔDIC of 10 or more rules out a model with the higher DIC [49]. Thus, strong statistical support for among-individual variation in trait plasticity was accepted if the DIC for the random slopes model was 10 or more lower than that for the random intercepts model. We report these ΔDICs between random intercepts and random slopes models in the results (§3).

Data for both day- and night-time movement activity (average swimming speed) were heavily zero-inflated, as cunner go dormant at cold temperatures [42,43] and also sleep at night, so about half or more of all fish did not move or leave their shelter during the behavioural trials across all temperatures and experiments, and thus ended up with a value of zero for movement activity. Thus, for the slow cooling experiment, Gaussian models were run for SMR, MMR and AS, whereas activity was analysed using both zero-altered poisson (ZAP) and categorical (binary) models; the former required rounding the activity data to the nearest integer, while the latter required transforming the activity data into binary data (0 = the fish did not move at all, 1 = the fish moved). We analysed the behavioural data from the slow cooling experiment using both ZAP and binary models to evaluate whether overall conclusions (support or not for random slopes, i.e. for plasticity variation) were affected by these data transformations. Results from both types of models are given in §3, but as both models produced similar results and conclusions, we analysed data from the fast cooling experiment as binary only and graphically present only the binary activity data overall. Data for food intake (number of pellets eaten) in the fast cooling experiment also were zero-inflated, with most fish eating either nothing (49% of observations) or their full ration of 10 pellets (34% of observations), and these data were therefore also analysed as binary (0 = the fish ate nothing, 1 = the fish ate something, with the majority of these eating everything).

The consistency of an individual's given trait relative to its conspecifics across repeated measurements was evaluated as trait repeatability, calculated from the most supported univariate model as the ratio of among-individual variance to among-individual variance plus residual variance [50]. Specifically, we calculated the conditional (context-specific) trait repeatability (*R*_C_) for each trait for each 1°C change in temperature, as detailed in Nakagawa & Schielzeth [50], in Killen *et al*. [51] and in the R script in the electronic supplementary material. For SGR, *R*_C_ changed with temperature in a skewed U-shaped manner (see §3) and so was calculated for every 0.1°C to more precisely identify the temperature where *R*_C_ was lowest. For the behavioural traits, *R*_C_ was calculated from the models with binary data only.

We used a multivariate model to investigate if there were correlations between traits in either intercepts or slopes in the slow cooling experiment—that is, if there was evidence for co-variation between the level of physiology and behaviour at the average temperature of 8°C (the model intercept, because temperature was centred around zero for analyses, meaning that raw 8°C was 0°C in the models), or for covariation between the thermal plasticities of physiology and behaviour (slope correlations). The multivariate model had the same fixed and random effects structures as for the univariate models described above, but with all of SMR, AS or MMR, SGR, day-time activity (binary) and night-time activity (binary) included as response variables in the same model. This approach is superior to extracting random intercepts and random slopes from separate models and correlating these, since the error associated with their estimation is not carried forward to the correlation analysis [52]. We ran two of these multivariate models—one with AS but not MMR and *vice versa*—since MMR and AS are nearly identical traits (MMR is AS plus a small value, SMR) and were very strongly correlated to one another, making the model unable to reliably handle both at the same time. Also, we only correlated plasticities (reaction norm slopes) of traits for which there was support for random slopes in the univariate models. For the fast cooling experiment, we investigated relationships between day-time activity and food intake (both binary) in a similar multivariate model, except with only the two traits as response variables (i.e. a bivariate model). Relationships between day-time activity from the slow and fast cooling experiments were also analysed in a bivariate model.

For all models, we used weakly informative parameter-expanded priors, with residual variance fixed at 1 for binary activity and food intake. For the slow cooling experiment, we sampled the posterior distribution 15 000 times by running the Markov chain for a total of 650 000 iterations (nitt) and sampling it every 40 iterations (thin) after discarding the first 50 000 iterations (burnin) for chain convergence. For the fast cooling experiment, we also sampled the posterior 15 000 times but doubled nitt, thin, and burnin to alleviate an issue with low effective sample sizes. See R script in the electronic supplementary material for exact prior and model structures. All correlations (cor) and repeatabilities (*R*_C_) are calculated from model variances and presented as posterior means along with their 95% CIs in square brackets.

## 3. Results

### (a) Slow cooling experiment (0.12°C/day)

**Figure 2.**
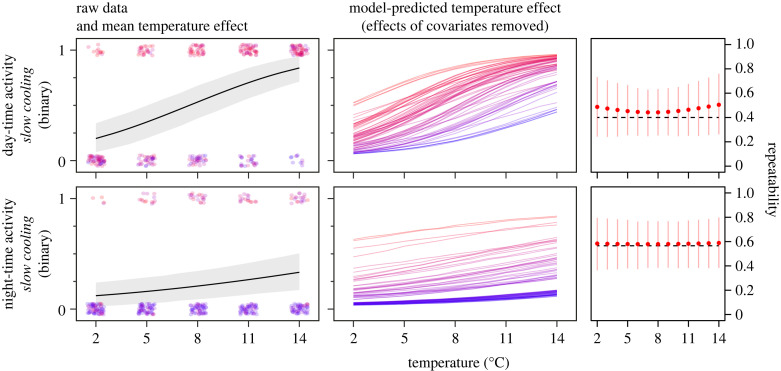
Day- and night-time activities of individual cunner (coloured dots and lines) cooled slowly from 14 to 2°C at a rate of 0.12°C/day. Activity data were originally collected as average swimming speeds (mm/s) but presented here after being converted into binary data (0 = fish did not move at all, 1 = fish moved) due to heavy zero-inflation. Panels show raw data (dots, jittered vertically and horizontally) with mean temperature effect (black line with grey 95% CI band), model-predicted temperature effects (thermal reaction norms) from the random slopes model after controlling for covariates, and repeatability estimates for day- and night-time activity. Dots and lines representing individual fish are coloured-coded within traits in translucent blue to red from the lowest to highest model-predicted trait values at 8°C, the model intercept temperature; darker regions are due to overlap. Note that we show model predictions from the random slopes models, despite the random intercepts models being most supported, since the predicted values (individual lines) would all be parallel for the random intercepts models, providing no visual information about the (low) degree of among-individual variation in trait plasticity. For repeatability, the red dots and red vertical lines are mean conditional repeatability estimates and their 95% CIs, respectively, for each trait, calculated from the random slopes models, while the dashed black horizontal lines are the mean repeatability estimates calculated from the most supported random intercepts models; we include the conditional repeatabilities here for consistency with figure 1.

Temperature had a significant overall effect on all physiological and behavioural traits (pMCMC < 0.005), so that cunner metabolic rates, growth and behaviours all decreased with decreasing temperature when cooled from 14 to 2°C at a naturally realistic rate of 0.12°C/day (figures 1 and 2). We also found strong evidence (ΔDIC > 10) for variation among individual cunner in the thermal sensitivity (plasticity) of their metabolic rates and growth rate; this was the case for all of SMR (ΔDIC = 92), MMR (ΔDIC = 30), AS (ΔDIC = 38) and SGR (ΔDIC = 50), as shown by the profound among-individual variation in thermal reaction norm slopes (figure 1). This among-individual variation in both metabolic and growth rate plasticity was highly predictable, as we also found strong and positive correlations between model-predicted reaction norm intercepts and slopes for all of SMR (cor = 0.835 [0.645, 0.997]), MMR (cor = 0.867 [0.654, 0.999]), AS (cor = 0.883 [0.690, 0.999]) and SGR (cor = 0.814 [0.562, 0.998]), meaning that individuals with relatively high metabolic or growth rates at the average temperature (intercepts) exhibited the overall greatest change (slopes) in these traits when the environmental temperature changed, compared to their low-metabolic-rate conspecifics.

We did not find any evidence for among-individual variation in behavioural plasticity of either day-time activity (ΔDIC_binary_ = 4.2, ΔDIC_ZAP_ = 1.9) or night-time activity (ΔDIC_binary_ = −0.12, ΔDIC_ZAP_ = 2.8), meaning that all individuals exhibited the same thermal sensitivity of these behavioural traits (figure 2).

All traits investigated here were significantly repeatable (figures 1 and 2), meaning that individuals were consistent in the relative level of a trait compared to their conspecifics across repeated measurements, but repeatability (*R*_C_) changed profoundly across temperatures for all metabolic rate traits and SGR due to the presence of among-individual variation in thermal plasticity, with highest repeatability at the warmest temperature of 14°C for all of SMR (*R*_C,14°C_ = 0.706 [0.606, 0.797]), MMR (*R*_C,14°C_ = 0.468 [0.309, 0.618]) and AS (*R*_C,14°C_ = 0.501 [0.350, 0.643]) and at the warmest mean temperature of 12.5°C for SGR (*R*_C,12.5°C_ = 0.535 [0.372, 0.688]). Repeatability decreased with temperature and approached zero at 2°C for all of SMR (*R*_C,2°C_ = 0.183 [0.023, 0.354]), MMR (*R*_C,2°C_ = 0.079 [0.005, 0.174]) and AS (*R*_C,2°C_ = 0.072 [0.003, 0.162]), with the lowest repeatability of SGR being at 5.6°C (*R*_C,5.6°C_ = 0.078 [0.004, 0.171]) due to thermal reaction norms for SGR crossing around this temperature (figure 1). Repeatability was the same across all temperatures for both day-time activity (*R*_C_ = 0.400 [0.207, 0.572]) and night-time activity (*R*_C_ = 0.563 [0.373, 0.757]) in the absence of among-individual variation in phenotypic plasticity (figure 2).

**Figure 3.**
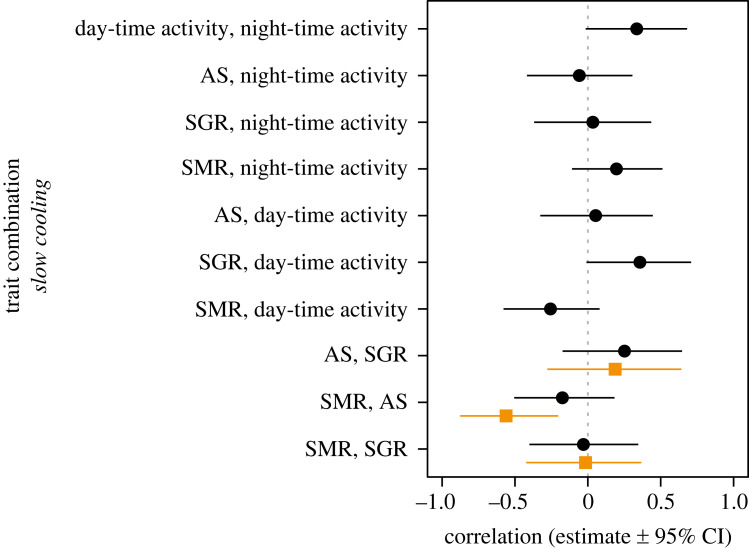
Pair-wise correlations between the physiological and behavioural traits of individual cunner cooled slowly from 14 to 2°C. Black dots and black horizontal lines are mean correlation estimates between trait intercepts (individual model-predicted trait values at the model intercept temperature of 8°C) and their 95% CIs, respectively, while orange squares and orange horizontal lines are mean correlation estimates between model-predicted slopes (plasticity) and their 95% CIs, respectively. Slope correlations were only done for traits with statistical support for random slopes (among-individual variation in phenotypic plasticity). Correlations are considered significant at the 95% probability level if the CIs do not cross zero (vertical dashed grey line). Note that we ran two separate multivariate models with either AS or MMR, as detailed in the main text, which produced very similar results, so we present only correlations from the model with AS here.

Our multivariate model of the physiological and behavioural traits showed a borderline significant positive intercept–intercept correlation (i.e. at the average temperature, 8°C) between individual day-time activity and SGR (cor = 0.357 [−0.009, 0.709]; pMCMC ≅ 0.055) (figure 3). We also found a borderline significant positive intercept correlation between day- and night-time activity (cor = 0.336 [−0.015, 0.681]; pMCMC ≅ 0.063) (figure 3). For correlations between trait slopes (i.e. phenotypic plasticity), we found that plasticity of SMR was negatively correlated with plasticity of both AS (cor = −0.562 [−0.887, −0.213]) and MMR (cor = −0.489 [−0.843, −0.005]) (figure 3), such that individuals with a high thermal sensitivity of SMR (steep reaction norm slope) had low thermal sensitivities of AS and MMR (shallow reaction norm slopes), and *vice versa*. No other traits were correlated in their plasticity (figure 3).

### (b) Fast cooling experiment (2°C/day)

**Figure 4.**
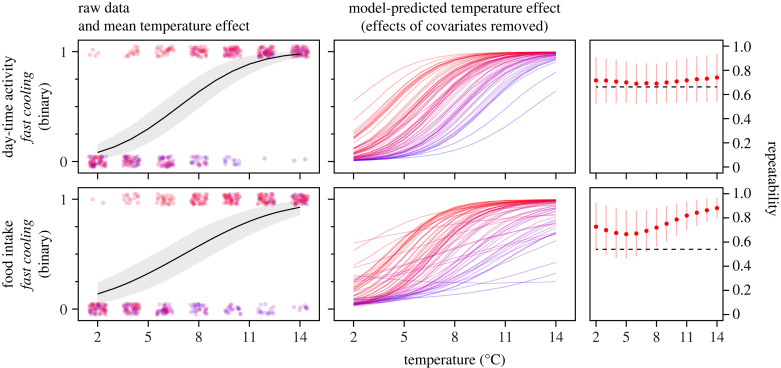
Day-time activity and food intake of individual cunner cooled relatively fast from 14 to 2°C at a rate of 2°C/day, for the same individuals as were used in the slow cooling experiment. Activity data were originally collected as average swimming speeds (mm/s) and food intake as number of pellets eaten, but both are presented here after being converted into binary data (0 = fish did not move or eat at all, 1 = fish moved or ate something). Panels show raw data (dots, jittered vertically and horizontally) with mean temperature effect (black line with grey 95% CI band), model-predicted temperature effects (thermal reaction norms) from the random slopes models after controlling for covariates, and conditional (context-specific) repeatability estimates for day-time activity and food intake. Dots and lines representing individual fish are coloured-coded within traits in translucent blue to red from the lowest to highest model-predicted trait values at 8°C, the model intercept temperature; darker regions are due to overlap. Note that we show model predictions from the random slopes models, which had circumstantial support, and since the predicted values (individual lines) would all be parallel for the random intercepts model, providing no visual information about the degree of among-individual variation in trait plasticity. For repeatability, the red dots and red vertical lines are mean conditional repeatability estimates and their 95% CIs, respectively, for each trait, calculated from the random slopes models, while the dashed black horizontal lines are the mean repeatability estimates calculated from the random intercepts models; we include the conditional repeatabilities here for consistency with figure 1 and due to the circumstantial evidence for random slopes for both day-time activity and food intake.

Only behaviour (day-time activity) and food intake were recorded in this follow-up experiment (using the same fish as in the slow cooling experiment), and both traits decreased significantly with decreasing temperature (pMCMC < 0.001; figure 4). Comparisons between random intercepts and random slopes models indicated that there was support for random slopes, i.e. among-individual variation in thermal sensitivity (reaction norm slopes) of food intake (ΔDIC = 58) and borderline for activity (ΔDIC = 5.3), but effective sample sizes from the random slopes models were low for both traits, indicating that our estimated parameter values were not independent and thus interpretations about among-individual plasticity variation should be made with caution. In line with this caveat, there was no support for co-variation in plasticity (thermal reaction norm slopes) of day-time activity and feeding (cor = 0.302 [−0.411, 0.915]), whereas the two traits were positively correlated at their intercept level (cor = 0.420 [0.126, 0.698]), meaning that more active individuals ate more than inactive ones at 8°C.

Repeatability calculated from the random intercepts model was relatively high and significant for both day-time activity (*R*_C_ = 0.663 [0.498, 0.806]) and food intake (*R*_C_ = 0.540 [0.380, 0.697]). Bearing the uncertainty of the random slopes model in mind, mean *R*_C_ estimates from these models across temperatures were higher than those from the random intercepts model, with a dip at intermediate temperatures for both day-time activity and feeding, but 95% CIs completely overlapped with the mean repeatability estimate from the random intercepts model for day-time activity and partially overlapped for food intake (figure 4).

### (c) Slow and fast cooling experiments combined

Day-time activity was the only trait shared by the slow and fast cooling experiments. We found a significant positive correlation between the intercepts of day-time activity from the two experiments (cor = 0.422 [0.108, 0.721]), meaning that individuals that were more or less active at 8°C during slow and naturally realistic cooling also were more or less active, respectively, during fast(er) cooling. Since there was no or only borderline support for random slopes of day-time activity in the slow and fast cooling experiments, respectively, correlation between trait plasticities was not evaluated.

## 4. Discussion

To investigate if plasticity in physiology and behaviour of individual animals (co)varies across environmental contexts, we repeatedly quantified the metabolic rates, growth rate and movement activity (voluntary swimming) of individuals of a marine fish, the cunner (*Tautogolabrus adspersus*), as they were cooled from 14 to 2°C, first gradually at a seasonally realistic rate of temperature change of 0.12°C per day (slow cooling experiment) and then at a faster rate of 2°C per day in a follow-up study performed on the same individuals after reacclimating them to 14°C (fast cooling experiment).

We found strong evidence for predictable among-individual variation in phenotypic plasticity of all of standard metabolic rate (SMR), maximum metabolic rate (MMR), aerobic scope (AS; MMR minus SMR) and specific growth rate (SGR) in the slow cooling experiment. Specifically, individuals with the highest metabolic rates or growth rates in the average environment (8°C here, the point around which we centred temperature in our models and thus where intercept variation is estimated) were those whose metabolic rates or growth rates were the most thermally sensitive; that is, they had the steepest thermal reaction norm slopes. These findings run opposite to those of a previous study on another fish species, the barramundi (*Lates calcarifer*), which found that individuals with the highest SMRs, MMRs or ASs were the least thermally sensitive when warmed acutely from 29 to 35°C [11]. These contrasting findings suggest that there could be variable and even opposite relationships between thermal reaction norm intercepts and slopes of individuals depending on biogeography (tropical barramundi *versus* temperate cunner), thermal change rate (acute for barramundi *versus* slow and seasonally realistic for cunner) and thermal change direction (warming for barramundi *versus* cooling for cunner). Indeed, work on insects (the cricket *Hophlosphyrum griseus*) has shown that individual thermal sensitivity (*Q*_10_) of SMR evaluated over the temperature range 17–27°C was negatively correlated with thermal sensitivity of SMR of the same individuals over the range 7–17°C (after two weeks of acclimation to each of the three temperatures in a randomized order); that is, individuals that were the most thermally sensitive across relatively warm temperatures were the least thermally sensitive across relatively cool temperatures [53].

We found no evidence for among-individual variation in behavioural plasticity (movement activity during day or night) in either the slow or fast cooling experiments and, consequently, no co-variation between physiological and behavioural plasticities. It is possible that the lack of among-individual variation in behavioural plasticity could be related to the heavy zero-inflation of the activity data, which prompted us to treat activity as binary (fish moved or not), potentially causing a loss of information about among-individual differences for the individuals that moved. However, rounding the activity data to the nearest integer for use in the ZAP models, rather than treating the data as binary, produced the same conclusion about lack of among-individual variation in behavioural plasticity. Thus, we believe that that lack of behavioural plasticity variation is real; however, it is possible that the finding is specific to winter-dormant fishes, such as our study species, as a highly conserved behavioural response to cooling would be consistent with an adaptive seasonal strategy involving profound inactivity.

Among the metabolic rate traits assessed in the slow cooling experiment, we found a negative relationship between plasticity of SMR and plasticity of AS or MMR, such that individuals with high thermal sensitivity of SMR (steep reaction norm slope) had low thermal sensitivities of AS and MMR (shallow reaction norm slopes), and *vice versa*. This interesting finding indicates the presence of a temperature-dependent trade-off between maintenance energy expenditure (SMR) and aerobic capacity (AS or MMR); individuals with the most thermally sensitive SMRs had relatively low aerobic capacities at warm temperatures but relatively high aerobic capacities at cold temperatures, whereas the opposite was the case for individuals with the least thermally sensitive SMRs. This suggests that the tissue-level and biochemical processes underlying resting (SMR) versus active (AS or MMR) metabolic rates vary in their thermal sensitivities, further suggesting that there may be trade-offs in the evolution of the thermal reaction norms for these floors and ceilings of aerobic metabolism, respectively (e.g. [37]).

While studies are scarce on individual (co)variation in thermal reaction norms, our finding of a negative correlation between the thermal sensitivity of SMR and AS or MMR across individuals is opposite to what has been found for salamanders (*Plethodon albagula*), where thermal sensitivity of SMR was positively correlated with that for MMR and unrelated to thermal sensitivity of AS over a temperature range from 10 to 25°C [54]. These contrasting findings indicate that co-variation in thermal sensitivity of metabolic traits may differ across taxa or, perhaps more likely, depends on the nature of the thermal change and respirometry trials—the present study used gradual and slow cooling from 14 to 2°C over 100 days, with SMR calculated from overnight (17–18 h long) respirometry trials, whereas Careau *et al*. [54] acutely exposed their salamanders to 10, 20, 15 or 25°C (in that order) for only 3 h before estimating SMR and MMR over the following 15 min, with 10 days between each temperature exposure. A negative correlation between plasticity of SMR and that of AS or MMR goes against the common belief that resting and maximum aerobic metabolic rates should be positively related—which they may be within a given environment (e.g. [55,56])—and emphasizes the importance of quantifying not only individual variation in key organismal traits but also individual variation in the phenotypic plasticity (reaction norm slopes) of such traits.

The absence of any correlations between plasticity of metabolic rates and plasticity of behaviours, as evaluated for the slow cooling experiment, shows that these key organismal traits are free to change independently of one another when environmental temperature changes at a seasonally realistic rate, at least in the winter-dormant species of fish investigated here. This contradicts the pace-of-life syndrome concept [21,22], which suggests that physiology and behaviour should be linked in a slow–fast continuum, with an active lifestyle correlating with high metabolic rates. This pace-of-life syndrome concept has recently been revisited due to equivocal evidence for it, with the recognition that (variation in) phenotypic plasticity in response to changes in ecological conditions is likely to affect and blur relationships between traits that otherwise were predicted to be related in constant environments [26]. While this notion aligns with the absence of co-variation in metabolic and behavioural plasticity found here across environmental contexts (i.e. across temperatures), the present study also provides mixed support for the existence of a pace-of-life syndrome *within* a given environment, as we did not find any evidence for co-variation between metabolic rates and activity at the reaction norm intercept level (8°C). We did, however, find a positive relationship between growth rate and day-time activity in the slow cooling experiment, as well as a positive relationship between day-time activity and food intake in the fast cooling experiment (food intake was only quantified in the fast cooling experiment), supporting pace-of-life syndrome predictions that more active individuals can grow faster in a given environment due to greater food intake [22]. A positive relationship between growth rate and movement activity has also been found for fish in the wild; perch (*Perca fluviatilis*) that were more active as adults, when the fishes' movements were tracked using acoustic telemetry in a lake, had been growing faster as juveniles, inferred from growth increments in the fishes' scales [57].

It is also possible that the lack of support for the pace-of-life-syndrome concept when investigating co-variation in metabolic and behavioural trait plasticities, as done here, could be due to species-specific strategies for responding to environmental change. The cunner used in the present study use winter-dormancy as a strategy for coping with the seasonal change into a cold and food-limited environment, which manifests as strong reductions in behavioural movement activity and food intake during cooling ([42]; present study), resulting in low or even negative growth rates (weight loss), as observed here. The absence of among-individual variation in thermal plasticity of movement activity for the cunner suggests that the strong reduction in activity during cooling is highly conserved within the species, with little room for variation among individuals. Although co-variation in individual metabolic and behavioural plasticities has received little previous attention, work on another fish species (brown trout, *Salmo trutta*) has shown that individuals that reduced their SMR the most across a period of decreasing food availability did not change or even increased their activity level relative to conspecifics [28]. The authors of that study interpret this negative correlation between these measures of metabolic and behavioural plasticities as among-individual variation in strategies for coping with food scarcity in the brown trout, a species that remains active at winter temperatures; a finding that also argues against a positive, slow–fast, relationship between metabolic rate and behaviour.

The profound among-individual variation in phenotypic plasticity of all metabolic rate traits and growth rate found here for the cunner meant that the among-individual differences in metabolic and growth rates (i.e. trait repeatability) changed drastically across temperatures, such that all of these physiological traits were significantly more repeatable at warmer than cooler temperatures. Since natural selection acts on variation between individuals, and repeatability generally sets the upper limit to heritability [50,58,59], this profound change in trait repeatability suggests that any selective forces acting on metabolic or growth rates will be highly context-dependent and strongest at warmer temperatures. These findings support those of Auer *et al*. [5], who showed that repeatability of metabolic rates is lower (by approx. 35%) for animals living under more variable field compared to stable lab conditions. The strong context-dependency of trait repeatability observed here for metabolic rates but not behaviour could possibly also help explain why heritability (*h*^2^) of metabolic rates generally is found to be relatively low (mean ± s.e. *h*^2^ = 0.19 ± 0.06; [39]) compared to that for behaviours (mean ± s.e. *h*^2^ = 0.31 ± 0.013; [60]), especially since the opposite would have been more intuitive, as behaviour is thought to be one of the most labile of all organismal traits [61] if unconstrained by trade-offs with other behavioural traits (e.g. behavioural syndromes; [62–64]) or by the underlying physiology supporting behaviour, such as metabolic rate [6,8]. Overall—while evolutionary inferences drawn from repeatability estimates are often justified [59]—the evolutionary implications of individual variation in metabolic rate ought to consider among-individual variation in trait plasticity and resulting context-specific trait repeatability.

## Data Availability

The datasets and R analysis script are available in the electronic supplementary material [65].
